# Magnetic Hysteresis in Nanocomposite Films Consisting of a Ferromagnetic AuCo Alloy and Ultrafine Co Particles

**DOI:** 10.3390/ma10070717

**Published:** 2017-06-28

**Authors:** Federico Chinni, Federico Spizzo, Federico Montoncello, Valentina Mattarello, Chiara Maurizio, Giovanni Mattei, Lucia Del Bianco

**Affiliations:** 1 Dipartimento di Fisica e Scienze della Terra and CNISM, Università di Ferrara, I-44122 Ferrara, Italy; federico.chinni@unife.it (F.C.); federico.spizzo@unife.it (F.S.); federico.montoncello@unife.it (F.M.); 2 Dipartimento di Fisica e Astronomia and CNISM, Università di Padova, I-35131 Padova, Italy; valentina.mattarello@student.unipd.it (V.M.); chiara.maurizio@unipd.it (C.M.); giovanni.mattei@unipd.it (G.M.)

**Keywords:** magnetic hysteresis, magnetic anisotropy, exchange interaction, nanocomposite material, SQUID magnetization measurements, micromagnetic modeling

## Abstract

One fundamental requirement in the search for novel magnetic materials is the possibility of predicting and controlling their magnetic anisotropy and hence the overall hysteretic behavior. We have studied the magnetism of Au:Co films (~30 nm thick) with concentration ratios of 2:1, 1:1, and 1:2, grown by magnetron sputtering co-deposition on natively oxidized Si substrates. They consist of a AuCo ferromagnetic alloy in which segregated ultrafine Co particles are dispersed (the fractions of Co in the AuCo alloy and of segregated Co increase with decreasing the Au:Co ratio). We have observed an unexpected hysteretic behavior characterized by in-plane anisotropy and crossed branches in the loops measured along the hard magnetization direction. To elucidate this phenomenon, micromagnetic calculations have been performed for a simplified system composed of two exchange-coupled phases: a AuCo matrix surrounding a Co cluster, which represents an aggregate of particles. The hysteretic features are qualitatively well reproduced provided that the two phases have almost orthogonal anisotropy axes. This requirement can be plausibly fulfilled assuming a dominant magnetoelastic character of the anisotropy in both phases. The achieved conclusions expand the fundamental knowledge on nanocomposite magnetic materials, offering general guidelines for tuning the hysteretic properties of future engineered systems.

## 1. Introduction

The dominant issue in the search for innovative magnetic materials is the creation of nanocomposite systems consisting of at least two different magnetic phases. This approach adds some degrees of freedom to single-phase materials and allows them to overcome their intrinsic limits. Some well-known examples are the Fe-rich [1] and the NdFeB-based [2] crystalline alloys composed of two different ferromagnetic phases so intimately mixed at the nanoscale as to be coupled by magnetic exchange interaction, which results in outstanding soft and hard magnetic properties, respectively.

For magneto-recording and spintronic applications, magnetic nanocomposite systems are fabricated in the form of films. For instance, the magnetic exchange interaction between a ferromagnetic layer and an antiferromagnetic one is efficiently exploited to tune the anisotropy of the former and, thus, to control the magnetization reversal process, which is a strategic goal in the construction of modern magnetoresistive devices [3,4,5]. For the same purpose, ferromagnetic soft-hard composites are among the most investigated systems: the inter-phase exchange coupling allows the hard phase anisotropy to be controlled both in strength and direction and favors a faster magnetization switching as well as a reduction of the switching field [6,7].

In layered materials, the substrate itself can be considered as a main component of the system, able to affect the magnetic behavior of the on-top magnetic film. Properly engineered architectures of substrate plus underlayers are employed to govern the film growth and the microstructure and, hence, the magnetic properties [8]. This is the case of Co-based crystalline films for recording applications, typically consisting of nanograins with a robust ferromagnetic character separated by weakly ferromagnetic boundaries, which modulate the intergrain magnetic interaction [9].

A different scenario is that in which a mechanical stress is produced in the magnetic film due to the bonding with the substrate [10], opening the possibility of exploiting the magnetoelastic coupling to tune the magnetic anisotropy [11,12,13]. Tailoring the anisotropy is crucial in the field of magnetic sensors where, moreover, flexible substrates are now often employed [14,15].

Indeed, the study of the magnetic behavior of nanocomposite materials does not stop drawing increasing interest because of their intriguing fundamental properties and prospective applications.

In this rich context, we have studied the magnetic properties of a set of three typical films made of Au and Co in different concentration ratios, grown by the magnetron sputtering co-deposition technique. By this method, a bimetallic compound of Au and Co, which are immiscible as bulk phases [16], is formed and the samples can be modeled as consisting of a prevalent ferromagnetic AuCo alloy and of segregated ultrafine Co particles, the two phases being well intermixed. It is worth noting that alloys and intermetallic compounds of Au with magnetic 3d elements are fascinating materials of great scientific interest for their magnetic, magneto-optical, magneto-plasmonic, and catalytic properties [17,18,19,20,21,22].

This article is fully focused on the magnetism of these AuCo samples, which exhibit a peculiar and unexpected hysteretic behavior, showing in-plane anisotropy and loops with crossed branches when measured along the hard magnetization direction. Micromagnetic calculations have been carried out to elucidate such a complex phenomenology. The results indicate that the main features of the hysteresis process can be qualitatively well reproduced assuming the existence of two different exchange-coupled ferromagnetic phases, identified with the AuCo alloy and the embedded Co particles, with almost orthogonal anisotropy axes. We will indicate specific requirements that the magnetic configuration of this composite system must fulfill in order for the described hysteretic behavior to be predicted or intentionally produced also in other nanocomposite magnetic materials. 

## 2. Experimental Methods

### 2.1. Production and Composition of the AuCo Samples

AuCo films have been deposited at room temperature on natively oxidized (100)-silicon substrates by the magnetron sputtering technique, using a custom built apparatus in which the metal targets are tilted at ~30° with respect to the axis normal to the sample plane, allowing a simultaneous deposition of the two metals. A rotating sample-holder has been employed, grounded to the deposition chamber, to favor a uniform coverage of the substrate. Argon has been the processing gas (working pressure = 5 × 10^−3^ mbar) and the deposition rate was ~1.5 Å/s. 

In this article, we address a set of three typical films, with nominal thickness ~30 nm and Au:Co concentration ratios of 2:1, 1:1, and 1:2, labeled as Au_2_Co_1_, Au_1_Co_1_, and Au_1_Co_2_, respectively; they have been coated in the same deposition batch with a SiO_2_ cap layer (~80 nm thick), in order to prevent environmental contamination and oxidation. A continuous Co film, with thickness (22 ± 1) nm and SiO_2_ cap layer, has been also deposited to be used as a reference sample.

**Figure 1:**
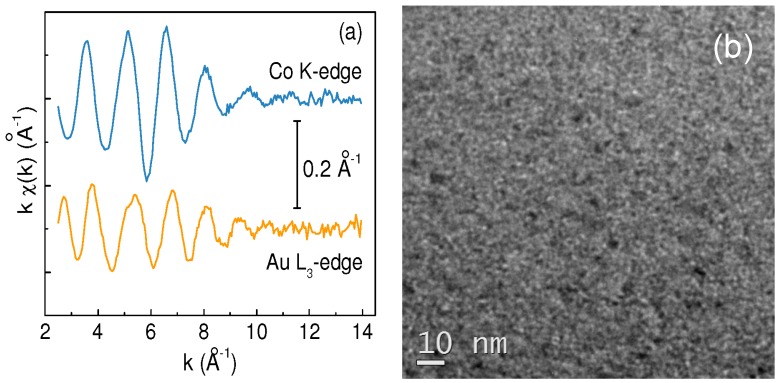
EXAFS spectra (**a**) and bright-field TEM image (**b**) for the Au_1_Co_2_ composition.

The film composition has been determined by Rutherford Backscattering Spectrometry (RBS) (Table 1). The RBS analysis together with SEM observations in cross-section mode also provided information on their thickness (Table 1). Information on the structure and composition of the films has been mainly acquired by Extended X-ray Absorption Fine Structure (EXAFS) investigation [23,24]. To provide a full description of this analysis is beyond the scope of the present research work. Here, we just summarize the principal conclusions. EXAFS experiments have been performed at both Au L_3_-edge and Co K-edge at T = 80 K (Italian Beamline BM08 of the European Synchrotron ESRF, Grenoble, France). The typical spectra for the Au_1_Co_2_ sample are displayed in Figure 1a.

In the spectra of all three films, the EXAFS analysis has evidenced a Au-Co interatomic correlation signal unambiguously revealing the formation of the AuCo alloy. A low first shell coordination number has been inferred, compared to bulk Au and Co, and no significant signal, except for that from the first intermetallic atomic coordination, has been detected. These results are consistent with an amorphous-like or poorly nanocrystalline structure. The AuCo alloy is the prevalent phase in all of the films. However, the EXAFS analysis has also indicated the presence of segregated cobalt in the form of structurally disordered particles (~2 nm in size) dispersed within the alloyed AuCo matrix, in general agreement with what was observed in similar samples [22]. The estimated volume fractions of the different phases in the films are reported in Table 1. Both the percentage of Co in the alloy and the fraction of segregated Co increase with decreasing the Au:Co ratio. A very small amount of segregated Au (~1 vol%) has been also considered in the fit of the EXAFS spectra of all three films.

Other structural investigation techniques are quite unable to provide more precise information. For instance, in Figure 1b we show a typical in-plane view by transmission electron microscopy in bright-field (BF-TEM) for the Au_1_Co_2_ composition (analysis performed with a Field emission FEI TECNAI F20 Super Twin FEG (S) TEM). The BF-TEM contrast is quite uniform with some black and white spots of a few nanometers in size: considering the abovementioned poorly crystalline nature of the films evidenced by the EXAFS analysis, the BF-TEM contrast is dominated by mass-contrast with negligible contribution from diffraction-contrast. Therefore, we can qualitatively interpret the BF-TEM image as arising from a composite film, made by some segregated Au-rich nanostructures (dark spots) and some Co-rich nanostructures (white spots), both embedded in a AuCo alloy, which constitutes the dominant phase. Similar conclusions can be drawn for the other investigated compositions.

### 2.2. Magnetic Methods

The magnetic properties of the AuCo films have been studied in the 6–300 K temperature range using a superconducting quantum interference device (SQUID) magnetometer (maximum applied field H = 50 kOe). For the SQUID analysis, the sputtered samples have been cleaved according to the crystallographic orientation of the substrate, so as to obtain pieces of about (5 × 5) mm^2^. The hysteresis loops have been corrected for the diamagnetic contributions of the Si substrate and SiO_2_ cap layer. The saturation magnetization M_S_ has been determined as the magnetic moment/sample volume. M_S_ of the reference Co film has been also measured at T = 6 K and 300 K.

Micromagnetic simulations have been carried out using the object-oriented micromagnetic framework (OOMMF) code from NIST [25]. In the following, to facilitate the comparison, loops measured along orthogonal in-plane directions and simulated loops are shown normalized to M_S_.

## 3. Results and Discussion

### 3.1. Sample Magnetization

Table 2 reports the values of M_S_ for the AuCo samples, measured at temperature T = 6 K and 300 K in H = 50 kOe. In the reference Co film, at T = 6 K M_S_ = (1250 ± 60) emu/cm^3^ and it does not depend appreciably on temperature in the investigated range, within the error; it is lower than the literature value of bulk Co (1446 emu/cm^3^ at T = 0 K), in general agreement with other results on thin Co films [26]. M_S_ increases with increasing the Co content in the films. One can easily realize that the M_S_ values are higher than the magnetization contributions provided by the Co particles (we have attributed the M_S_ of the reference Co film to the segregated cobalt for this calculation). Hence, in all the samples, the AuCo alloy has a net magnetization, namely is ferromagnetic. Based on the volume fractions in Table 1, calculated values of saturation magnetization for the AuCo alloy at T = 6 K are ~230 emu/cm^3^ for sample Au_2_Co_1_, ~280 emu/cm^3^ for Au_1_Co_1_, and ~500 emu/cm^3^ for Au_1_Co_2_ (the relative error associated to these values ranges between 20% and 35%).

### 3.2. Measured Hysteretic Properties

**Figure 2:**
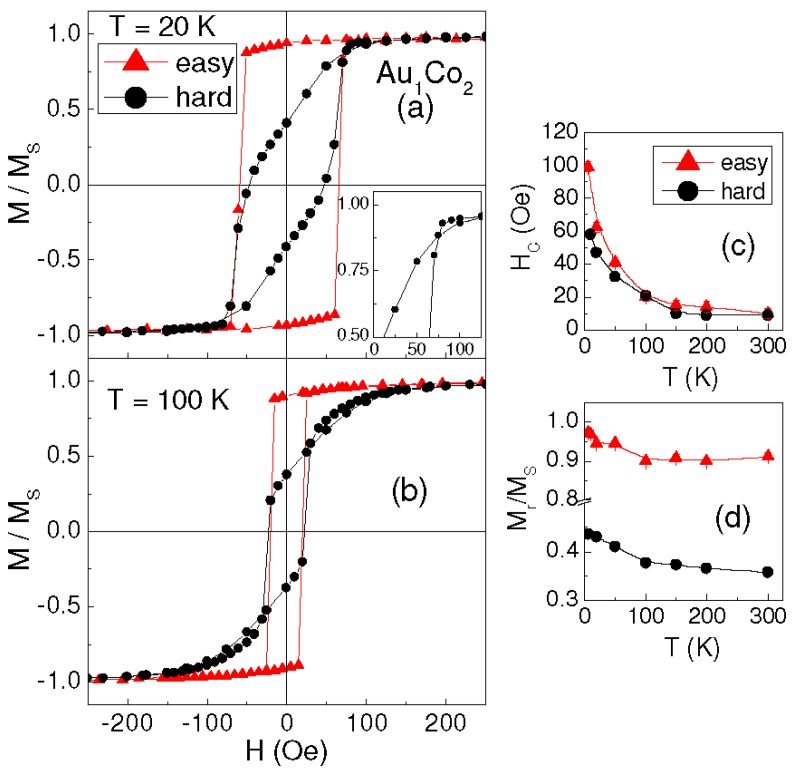
In-plane hysteresis loops measured on sample Au_1_Co_2_ at T = 20 K (**a**) and T = 100 K (**b**), along two orthogonal directions indicated as easy (triangles) and hard (circles). The curves are normalized to the saturation magnetization M_s_. The inset of frame (a) is an enlarged view of the hard axis loop, highlighting the crossing of the two branches. Coercivity H_c_ (**c**) and squareness ratio M_r_/M_S_ (**d**) vs. T; the values measured along the easy and hard axes are displayed (in some cases, the error bar is smaller or comparable to the symbol size; solid lines are guides for the eye).

First, let us consider the sample Au_1_Co_2_. Figure 2a shows the loops measured at T = 20 K along two in-plane orthogonal directions corresponding to the sides of the sample. Along one direction, the loop appears highly squared, with the ratio between the remanent magnetization and saturation magnetization M_r_/M_S_ ~ 0.97; the loop measured in the orthogonal direction has a lozenge shape with a smaller squareness ratio (M_r_/M_S_ ~ 0.41) and shows a very peculiar feature, namely the crossing of the two branches (Figure 2a, inset). Hence, the magnetic behavior is clearly anisotropic and the direction corresponding to the high-remanence loop is a preferential magnetization axis, that we conventionally indicate as the easy axis; accordingly, the orthogonal direction is indicated as the hard axis. The same hysteretic behavior is observed at higher temperature, up to 300 K (the loops for T = 100 K are displayed in Figure 2b). The curves of H_C_ and M_r_/M_S_ vs. T, measured along the easy and hard axes, are shown in Figure 2c,d.

**Figure 3:**
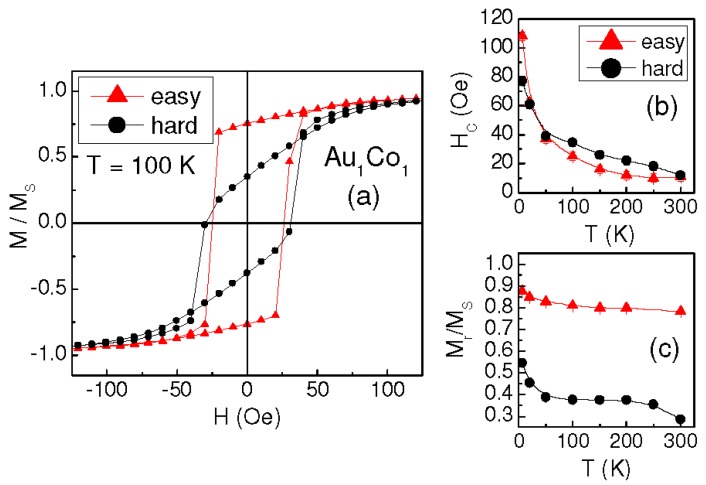
(**a**) In-plane hysteresis loops measured on sample Au_1_Co_1_ along the easy and hard orthogonal axes at T = 100 K (normalized to the saturation magnetization M_s_), Coercivity H_c_; and (**b**) squareness ratio M_r_/M_S_; (**c**) vs. T, along the easy and hard axes.

Sample Au_1_Co_1_ exhibits a very similar magnetic behavior. Typical hysteresis loops, measured along the orthogonal directions, are displayed in Figure 3a (T = 100 K); H_C_ and M_r_/M_S_ vs. T are reported in Figure 3b,c. In this sample we can also distinguish an easy magnetization axis, corresponding to the direction along which the high-remanence loops are measured, and the loops measured along the hard axis present crossed branches. 

**Figure 4:**
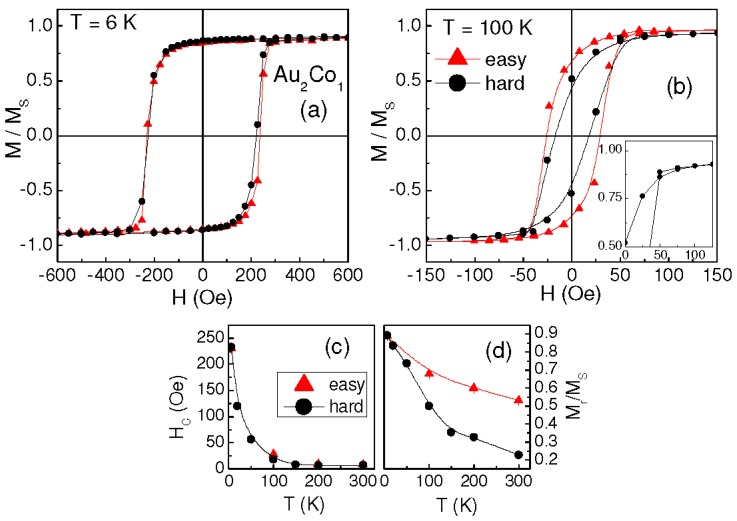
In-plane hysteresis loops measured on sample Au_2_Co_1_ along the easy and hard orthogonal axes at T = 6 K (**a**) and T = 100 K (**b**) (normalized to the saturation magnetization M_s_). Inset of frame (b): enlarged view of the hard axis loop. Coercivity H_c_ (**c**) and squareness ratio M_r_/M_S_ (**d**) vs. T along the easy and hard axes.

**Figure 5:**
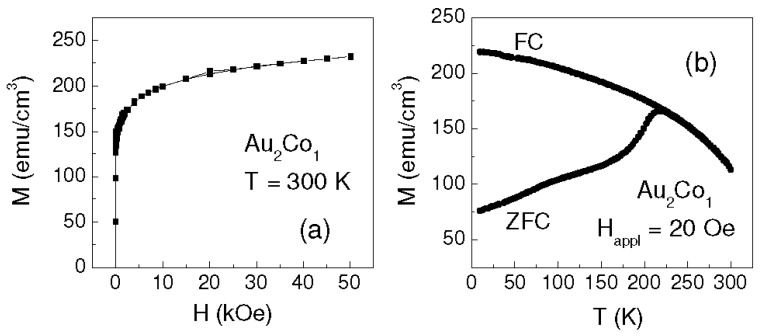
(**a**) First quadrant of the loop measured on sample Au_2_Co_1_ at T = 300 K; (**b**) Zero-field-cooling (ZFC) and field-cooling (FC) magnetization as a function of temperature, measured on Au_2_Co_1_ in an applied magnetic field H_appl_ = 20 Oe.

As for sample Au_2_Co_1_, the loops measured at 0° and 90° at T = 6 K are shown in Figure 4a. In this case, the film appears isotropic in the plane, but it turns to anisotropic with rising temperature. In fact, at T = 100 K, hysteretic properties similar to those found in Au_1_Co_2_ and Au_1_Co_1,_ even if less pronounced, are observed (Figure 4b), which persist at higher temperature. H_C_ and M_r_ vs. T are shown in Figure 4c,d. At the lowest temperature, H_C_ is higher than in the other investigated samples and it strongly decreases with increasing T especially up to ~100 K (there is no substantial difference in H_C_ along the easy and hard magnetization directions); M_r_ exhibits a similar decreasing trend (the values measured along the easy axis are obviously larger than those measured along the hard axis). Hence, the magnetic behavior of sample Au_2_Co_1_ is somewhat different compared to the other films and, in this respect, a remarkable difference is also a clear non-saturation tendency of the loops, which becomes more pronounced with increasing temperature (hence, for this sample, the values of M_S_ in Table 2, taken as those measured in H = 50 kOe, may be slightly underestimated; however, the saturation magnetization values will lie within the indicated error bar, reasonably). This effect is well visible in Figure 5a showing the first quadrant of the loop at T = 300 K (at high field, the trend of M vs. H does not visibly depend on the measurement direction). It should also be noted that a reduction of M_S_ of ~30% is experienced with increasing the temperature from 6 K to 300 K, whereas it is not larger than 8% in the other samples (Table 2).

In this class of samples, the magnetic exchange coupling of the Co particles with the surrounding matrix is expected to result in an increase of their effective magnetic size, with respect to the structural size. Moreover, since the fraction of Co particles in the films increases with decreasing the Au:Co ratio (Table 1) and their interdistance reduces and possibly vanishes, the exchange interaction may be transmitted to neighboring particles through the ferromagnetic matrix, leading to the formation of magnetically coupled clusters (i.e., aggregates of Co particles). 

However, in Au_2_Co_1_ the segregated cobalt is just a small fraction (~10 vol%) and the AuCo alloy has a weaker ferromagnetism compared to the matrix in the other films, being richer in Au. Hence, we hypothesize a weaker magnetic coupling between the Co particles and the AuCo matrix and between neighboring Co particles in this sample, compared to Au_1_Co_2_ and Au_1_Co_1_, which results in Co clusters with smaller magnetic size. This can account for the larger H_C_ of Au_2_Co_1_ at T = 6 K (~235 Oe), compared to the other films.

With increasing temperature, the strong thermal dependence of M_S_ and the marked non-saturation tendency of the hysteresis loops suggest that the smallest Co clusters undergo a magnetic relaxation process, similar to superparamagnetism [27]. In order to better elucidate this point, the magnetization M has been measured for increasing temperature in the 6–300 K range in H_appl_ = 20 Oe, after cooling the sample from room temperature down to 6 K both without an external field (zero-field-cooling, ZFC) and in H_appl_ (field-cooling, FC).

The result is shown in Figure 5b: a magnetic irreversibility effect, i.e., a difference between FC and ZFC magnetization, is clearly observed from T = 6 K up to ~215 K, confirming our hypothesis of magnetically relaxing Co clusters [27,28]. Accordingly, H_C_ reduces strongly with increasing temperature, especially in the 6–100 K range (H_C_ ~ 20 Oe at T = 100 K, Figure 4c). A similar, even if less marked, decrease of H_C_ at low temperature is also visible in Au_1_Co_2_ and Au_1_Co_1_ (Figure 2c and Figure 3b). Hence, we cannot exclude the possibility that a very small fraction of relaxing Co clusters also exists in these two samples, although the coercivity trend would be the only appreciable hint of this. Similar values of H_C_ are measured in the three samples for T ≥ 100 K. This suggests that, above this temperature, the magnetic behavior of Au_2_Co_1_ is also ruled by the AuCo matrix and by the fraction of the non-relaxing Co clusters and, for this reason, hysteretic properties emerge similar to those observed in the other samples.

### 3.3. Micromagnetic Simulations of the Hysteresis Loops

We have ascribed the observed hysteretic behavior, especially well visible in the samples Au_1_Co_2_ and Au_1_Co_1_, to the coexistence of two different magnetic phases, that we have identified with the AuCo matrix and the Co clusters. The two phases are intimately mixed and the magnetization process is expected to be governed by the subtle interplay between the strength and directions of their magnetic anisotropy, strength of the exchange interaction, and the external applied field. Since most of these parameters cannot be experimentally determined and because of the inherent complexity of the system, to achieve a full comprehension of the magnetization process in the investigated samples is a quite difficult task. We have obtained valuable information by considering a simplified model of a ferromagnetic two-phase system and performing micromagnetic calculations by OOMMF. OOMMF employs the finite difference method, which requires discretization of a chosen geometry over a grid of identical prism-cells in which the magnetization is supposed to be uniform. 

**Figure 6:**
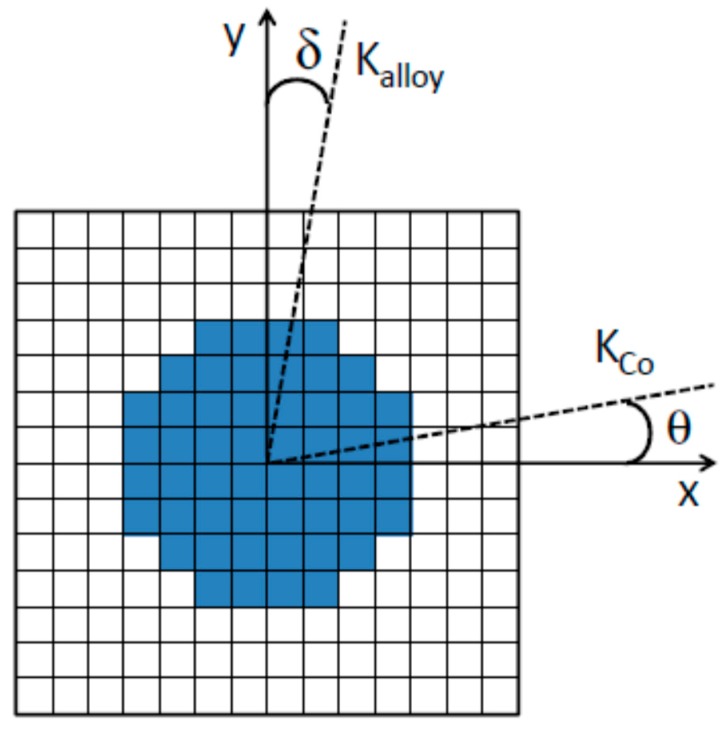
Scheme of the system simulated in the micromagnetic analysis. The squared element represents the AuCo matrix and the central disk represents a Co cluster. In this geometry, the volume fraction occupied by the Co cluster is 26.5%. The dotted lines are the anisotropy directions for the two phases: the Co anisotropy (K_Co_) forms an angle θ with the x axis and the AuCo anisotropy (K_alloy_) forms an angle δ with the y axis. See the text for details.

In our case, we have considered the geometry shown in Figure 6: a squared element hosting a circular disk at the center, both discretized using cells with a basis of (1.25 × 1.25) nm^2^ and height of 10 nm. In particular, the squared element consists of 14 × 14 cells and, hence, its area is 306.25 nm^2^ in the x-y plane and the thickness along the z direction is 10 nm. The central disk is also 10 nm-thick and occupies a volume corresponding to 26.5% of that of the squared element. The simulations have been carried out using 2D periodic boundary conditions, which means that the geometry in Figure 6 is periodically replicated in the whole x-y plane so as to simulate an infinite film. Hence, the central element represents a Co cluster (i.e., an aggregate of smaller Co particles) of 10 nm in size and the squared one represents the portion of surrounding AuCo matrix. The precise definition of such a system has been dictated by a number of concomitant factors. The Co cluster shape is cylindrical and the diameter is equal to the height in order to increase the symmetry of the structure preventing, at the same time, shape anisotropy effects and keeping the computation time under control; the choice of the 10 nm size is strictly intertwined to the dimension of the prism-cells. In fact, to avoid the occurrence of fictitious effects, the side of the cells has been set so as to fulfill two main requirements: (i) to smooth the jagged perimeter of the cylinder and (ii) to manage an even number of cells, preventing any artificial symmetry-breaking in the simulations. Moreover, we demanded that the relative volume fractions of the cylindrical and squared elements approximately corresponded to the volume fractions of the Co and AuCo phases in Au_1_Co_1_ (Table 1).

Coherently with the data for Au_1_Co_1_ reported in Section 3.1, we have assigned saturation magnetization values M_S_alloy_ = 280 emu/cm^3^ to the AuCo matrix and M_S_Co_ = 1250 emu/cm^3^ to the Co cluster. The magnetic exchange stiffness for Co is A_Co_ = 3 µerg/cm, corresponding to the literature value (provided by the OOMMF database [25]), whilst A_alloy_ was arbitrarily set to a value one order of magnitude smaller to take account of the Co dilution in the alloyed phase [29]. With this choice of A_alloy_ the ferromagnetic exchange length of the AuCo phase L_alloy_ = (A_alloy_/K_alloy_)^1/2^ is ~3 nm. This implies that, given the geometry in Figure 6, the matrix does not transmit the exchange interaction to neighboring Co clusters because L_alloy_ is shorter than the Co clusters’ interdistance [30].

The stiffness of the exchange interaction between the two phases has been set to 1.6 µerg/cm, i.e., intermediate between A_Co_ and A_alloy_. The anisotropy coefficients are K_Co_ = 5 × 10^6^ erg/cm^3^ for the Co element, corresponding to the literature value [31], and K_alloy_ = 2.4 × 10^6^ erg/cm^3^ for the AuCo alloy, about half of the former. We have considered that the anisotropy axes of both phases lie in the x-y plane as schematized in Figure 6: the Co anisotropy direction is defined by the angle θ it forms with the x axis whereas the AuCo anisotropy direction is defined by the angle δ it forms with the y axis. 

We have carried out simulations of hysteresis loops measured by applying the magnetic field H along the x and y axes; they will be shown as normalized, in ordinate, to the saturation magnetization and, in abscissa, to the anisotropy field of the matrix H_K_ = 2K_alloy_/M_S_alloy_.

**Figure 7:**
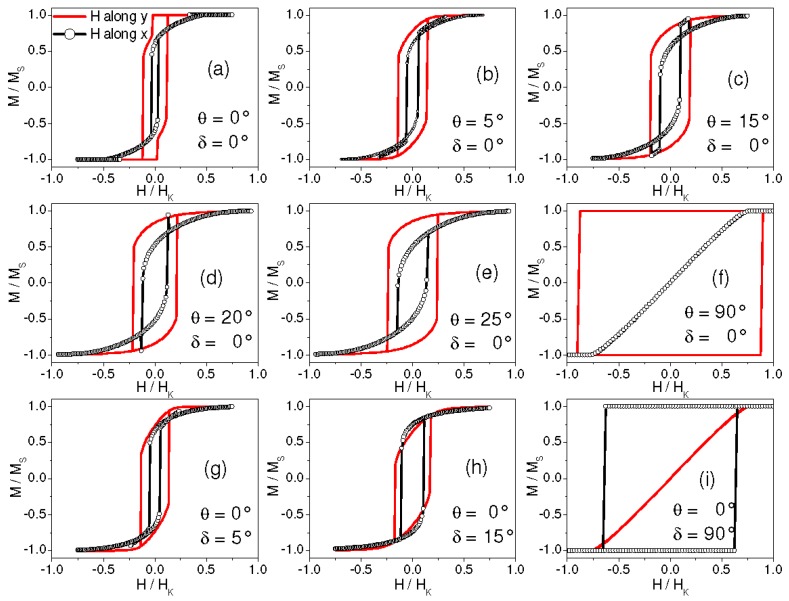
(**a**–**i**) Simulated hysteresis loops obtained for the indicated values of θ and δ, with magnetic field H applied along the y axis (continuous line) and the x axis (line with circles). H values are normalized to the anisotropy field H_K_ of the AuCo matrix.

In a first set of simulations, we have set δ = 0° (namely, the AuCo anisotropy direction is along y) and θ has been varied between 0° and 90°. Selected results are shown in Figure 7a–f: for each θ, the loops measured along y and along x are displayed. At all θ values an anisotropic hysteretic behavior is observed and the high-remanence loop is measured along the y axis, namely along the anisotropy direction of the AuCo matrix. Hence, we indicate the y axis as the easy magnetization axis of the system. For θ ≠ 90°, non-zero remanence is measured along the hard magnetization direction, i.e., along x. For θ = 90°, namely when K_alloy_ and K_Co_ are parallel, the loop measured along y is perfectly squared whereas no hysteresis is observed in the x direction (Figure 7f). This behavior is very close to that modeled by Stoner and Wohlfarth for a single-domain magnetic element with uniaxial anisotropy [32]. For small θ values, the loops measured along x show a crossing of the branches. The effect is well visible in Figure 7 for θ = 5°, 15°, and 20° (the best similarity with the experimental results is obtained for the smallest θ, actually). No crossing is observed at higher θ (Figure 7e). The same results are obtained for negative values of θ, namely imposing a negative slope to the K_Co_ axis. Then, we have studied the case of θ = 0° and variable δ. Selected results are shown in Figure 7g–i: for δ = 5°, the loop with higher remanence is measured along y and crossed branches are found in the loop along x; for δ = 15° the highest remanence is measured in the x direction (the loops have similar remanence actually) and no branch-crossing is visible; as expected, for δ = 90°, a Stoner–Wohlfarth type behavior is produced.

Hence, key ingredients for observing anisotropic hysteretic behavior and the branch-crossing effect are that the AuCo matrix has a well-defined anisotropy direction (the y axis, in our reference frame in Figure 6) and that the Co clusters’ anisotropy lies almost orthogonally to that of the matrix, i.e., it is just slightly misaligned with respect to the x axis.

Now, the point is whether these requirements can be fulfilled in real samples. The possibility that the anisotropy ruling the magnetic behavior has a magnetocrystalline origin appears unlikely. In fact, the AuCo alloy presents a poorly crystalline structure, which implies a distribution of locally varying easy axes, possibly resulting in a vanishingly small averaged anisotropy [33]. In this view, we are not able to account for the appearance of a net uniaxial magnetocrystalline anisotropy of the matrix. As for the Co clusters, if the prevalent type of anisotropy was the magnetocrystalline one, they should possess a preferential crystallographic orientation in the plane of the film so that their anisotropy vectors would lie within a cone of limited angular width. However, the structural analyses exclude an in-plane texturing of the Co phase [23].

We must consider a different scenario and we propose that both phases have dominant magnetoelastic anisotropy. The magnetoelastic anisotropy energy is expressed by the relation E = 3/2λ_S_σsin^2^φ, where λ_S_ is the magnetostriction, σ is the mechanical stress, and φ is the angle between the stress axis and the magnetization [31]. Cobalt is a magnetostrictive material and the AuCo alloy is likely to possess the same property (no information can be found in literature on this point). As for σ, it is well known that all films have a state of residual stress due to extrinsic factors (typically, the mismatch in the thermal expansion coefficients of the film and of the substrate) and to intrinsic factors (growth processes, grain structure, substitutional or interstitial impurities) [10]; in the case of composite films, lattice and thermoelastic mismatch among the different phases must be also taken into account [34,35]. Several of these factors may operate simultaneously so that the final stress distribution in the film results from their balance. Despite the fact that most of the models on the mechanical properties of film-substrate systems, such as the well-known Stoney theory [36], assume a spatial stress uniformity, a non-uniform stress distribution is much more likely to occur in practice [37] due, for instance, to the deviation between the ideal isotropic properties of the substrate and the real properties. A non-uniform stress distribution may be settled in our films, which implies that the average stress along two orthogonal directions may be different [10,38,39]. Although, at present, we are not able to unambiguously account for a non-uniform stress state in our samples, the observation that easy and hard magnetization axes are along the sample sides in all the three cases suggests that the stress distribution originates from the reproducible intervention of precise factors, presumably connected with the silicon substrate and/or with the production procedure of the samples. The item certainly deserves further attention also in the perspective of achieving a control of the stress state and thus governing the magnetoelastic anisotropy.

A small difference between the stresses acting along y and along x on AuCo (σ_y_alloy_ and σ_x_alloy_, respectively) and Co (σ_y_Co_, and σ_x_Co_) is sufficient to generate the inherent anisotropic character that emerges in the hysteretic magnetic behavior, depending on the interplay between their strength and sign and λ_S_. Two main pictures can be drawn. In the first one, the Co and AuCo alloy have magnetostriction constants of different signs. Since Co has negative λ_S_ [31], a positive λ_S_ is assigned to AuCo. We hypothesize that along the y axis the two phases are subjected to a stress with the same sign—taken as positive to preserve the coherence with the coordinate system adopted in Figure 6—and that σ_y_alloy_ > σ_x_alloy_ and σ_y_Co_ > σ_x_Co_. In this way, the anisotropy axis of the AuCo matrix is along the y direction and the anisotropy of the Co clusters preferentially lies along the x direction. In the second picture, both Co and AuCo have negative λ_S_. We hypothesize that, along the y axis, a negative stress acts on AuCo and a positive stress acts on Co and that |σ_y_alloy_| > |σ_x_alloy_| and σ_y_Co_ > σ_x_Co_. In this way too, the anisotropy directions for the AuCo matrix and for the Co clusters are along y and x, respectively. In this framework, the required small misalignment of K_Co_ with respect to the x axis, may be accounted for in terms of the competition between the dominant magnetoelastic anisotropy and the magnetocrystalline anisotropy, presumed to act on the Co clusters along random directions.

**Figure 8:**
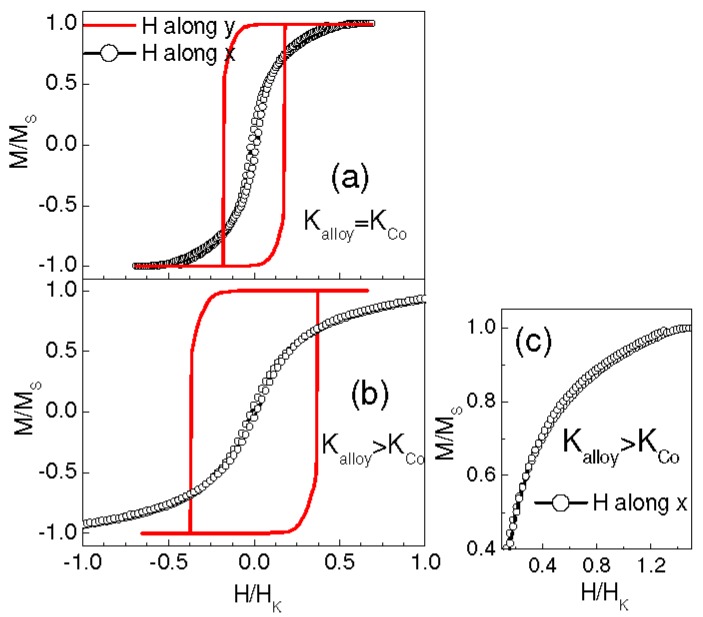
Simulated hysteresis loops obtained for δ = 0° and θ = 5° with magnetic field H applied along the y axis (line) and the x axis (line with circles). In (**a**) K_alloy_ = K_Co_ = 2.4 × 10^6^ erg/cm^3^; in (**b**) K_alloy_ = 5 × 10^6^ erg/cm^3^ and K_Co_ = 2.4 × 10^6^ erg/cm^3^; Frame (**c**) is an enlarged view of the loop along x as shown in (**b**).

Hence, by assuming that the anisotropy of AuCo and Co has a prevalent magnetoelastic character, different situations can be envisaged, which fulfill the requirements indicated by the micromagnetic study. By the light of this statement, our initial choice of setting K_Co_ to the magnetocrystalline anisotropy of bulk cobalt may appear not appropriate (also in consideration of the poor crystallinity of the Co phase in real samples) and also the choice of K_alloy_ has been arbitrary. Calculations carried out using different values of K_Co_ and K_alloy_, but in the same relationship, produce similar results, indicating that the outcomes do not strictly depend on the assigned anisotropy coefficients. Moreover, Figure 8a shows the loops obtained imposing both to the AuCo matrix and to the Co element the same anisotropy (K_alloy_ = K_Co_ = 2.4 × 10^6^ erg/cm^3^), whereas in Figure 8b,c, K_alloy_ = 5 × 10^6^ erg/cm^3^ and K_Co_ = 2.4 × 10^6^ erg/cm^3^ (namely, compared to the previous simulations, the anisotropy values for AuCo and Co have been exchanged). In both cases, we have set δ = 0° and θ = 5°, as in Figure 7b. The main features of the hysteretic behavior are still visible. However, the remanent magnetization measured along x is substantially smaller than in Figure 7b, a characteristic that worsens the qualitative agreement with the experimental results.

**Figure 9:**
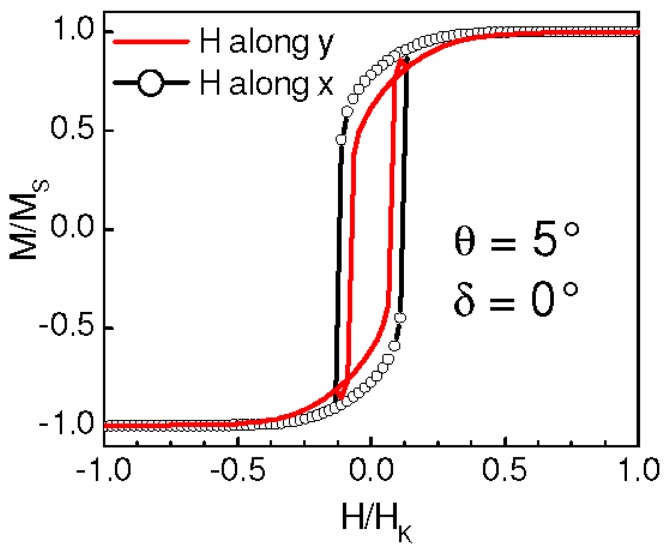
Hysteresis loops obtained by simulating a system slightly different from that in Figure 6, namely in which the volume fraction of the Co cluster is higher (~35%). Loops are calculated for θ = 5° and δ = 0° with magnetic field H applied along the y axis (line) and the x axis (line with circles).

Finally, we have addressed the case of a sample with a higher Co content. For this purpose, we have reduced the extension of the squared element drawn in Figure 6, down to a size of 12 × 12 cells. In this way, the volume occupied by the Co inclusion is ~35%, corresponding to the fraction that exists in sample Au_1_Co_2_ (Table 1). Accordingly, we have assigned a magnetization M_S_alloy_ = 500 emu/cm^3^ to the AuCo matrix (see Section 3.1). All the remaining parameters are unchanged with respect to the simulations in Figure 7. In particular, in Figure 9 we present the results obtained for δ = 0° and θ = 5°. In this case, the high-remanence loop is measured along x and the cross-branching effect is visible in the loop along y. Hence, the easy and hard axes are exchanged with respect to Figure 7b, a situation that cannot be distinguished by experiments on the real samples.

Regarding sample Au_2_Co_1_, the simulation approach used for the other two samples is affected by the difficulty of assessing the fractions of non-relaxing and relaxing Co clusters, which should actually be treated as two different magnetic phases. However, the loops calculated for a smaller Co element in Figure 6 (corresponding to a volume fraction of ~10%, Table 1) and with the appropriate value of M_S_alloy_, show basic characteristics close to those found for sample Au_1_Co_1._

## 4. Conclusions

We have studied the magnetic properties of AuCo films, with different atomic ratios of the constituent metals, prepared by co-sputtering deposition. The samples consist of an almost amorphous AuCo alloy in which nanosized Co particles are dispersed. Indeed, the magnetic analysis can be a valuable tool to gain structural information, hardly accessible otherwise. Our study has nicely revealed the composite magnetic structure of the samples, disclosing, in particular, an unexpected hysteretic behavior, characterized by in-plane anisotropy and branch-crossing in the loop measured along the hard magnetization direction. In fact, these characteristics have been satisfactorily reproduced by micromagnetic calculations relative to a simplified system representing two exchange-coupled ferromagnetic phases—a matrix embedding a different element—with almost orthogonal anisotropy axes. The two phases have been identified with the AuCo alloy and with a Co cluster. Different scenarios have been discussed in order to explain how the requirement of orthogonal anisotropy axes can be fulfilled. In particular, we have assumed a dominant magnetoelastic character of the anisotropy of the two phases, which implies that both of them must be magnetostrictive and subjected to a non-uniform stress distribution. In the perspective of creating novel magnetic architectures, for which flexible substrates are now more and more often employed, the latter condition can be intentionally induced, so as to obtain a control of the anisotropy and hence of the loop shape, particularly of the remanence state and of the approach to saturation. In this view, the particular hysteretic features that we have described could become the fingerprint of the existence of (almost)-orthogonal anisotropy axes.

Samples Au_1_Co_1_ and Au_1_Co_2_ have been preferably addressed in the micromagnetic study. The simulation approach for Au_2_Co_1_ is made complicated by the difficulty in estimating the fractions of the stable Co clusters and of the magnetically relaxing ones, which strongly affect the overall magnetic behavior, especially for T < 100 K.

The micromagnetic analysis has succeeded in attaining general guidelines regarding how the observed hysteretic properties may originate in the investigated material. The obtained results contribute to expanding the fundamental comprehension of nanocomposite magnetic systems and offer interesting implications for the creation of future composite materials.

## Figures and Tables

**Table 1:** Column 1: labels of the samples. Columns 2 and 3: Au and Co content in the films. Column 4: thickness of the films. Columns 5–7: volume fractions of the AuCo alloy, with its composition, and of segregated Co and Au (to convert atomic fractions to volume fractions, elemental atomic weights and densities have been used).

Sample	Composition		Thickness (nm)	Volume Fractions		
	Au (at%)	Co (at%)		Alloy (vol%)	Co (vol%)	Au (vol%)
Au_2_Co_1_	69 ± 1	31 ± 1	30 ± 2	Au_80_Co_20_ 89 ± 7	10 ± 3	1.0 ± 0.8
Au_1_Co_1_	49 ± 1	51 ± 1	28 ± 1	Au_73_Co_27_ 74 ± 10	25 ± 5	1.0 ± 0.8
Au_1_Co_2_	33 ± 1	67 ± 1	28 ± 1	Au_57_Co_43_ 64 ± 9	35 ± 5	1.0 ± 0.9

**Table 2:** Column 1: labels of the samples. Column 2: saturation magnetization (M_S_) measured at temperature T = 6 K in H = 50 kOe. Column 3: M_S_ at T = 300 K. The main error source is the uncertainty of the sample thickness.

Sample	M_S_ at T = 6 K (emu/cm^3^)	M_S_ at T = 300 K (emu/cm^3^)
Au_2_Co_1_	325 ± 22	232 ± 15
Au_1_Co_1_	523 ± 20	480 ± 18
Au_1_Co_2_	755 ± 28	733 ± 27

**Figure A1:**
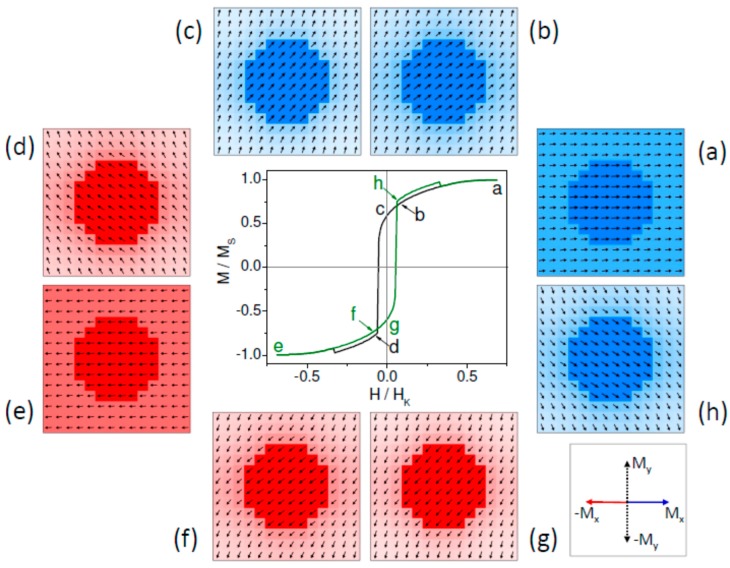
Central frame: hysteresis loop calculated for δ = 0°, θ = 5° and H applied along the x axis (already shown in Figure 7b); the descending branch (from positive to negative saturation) is the black line, whereas the ascending branch (from negative to positive saturation) is the green line (in the regions where the two branches are superposed, only the green line is visible). The magnetization maps corresponding to the indicated points of the loop are shown: they are labelled with letters from (**a**) to (**h**). The key frame on the bottom-left corner indicates that the blue and red colors in the maps designate the sign of the projection of the magnetization along the x axis, M_x_ (positive and negative, respectively); no specific color is assigned to the magnetization projection along the y axis, M_y_. See the text for further explanation.

**Figure A2:**
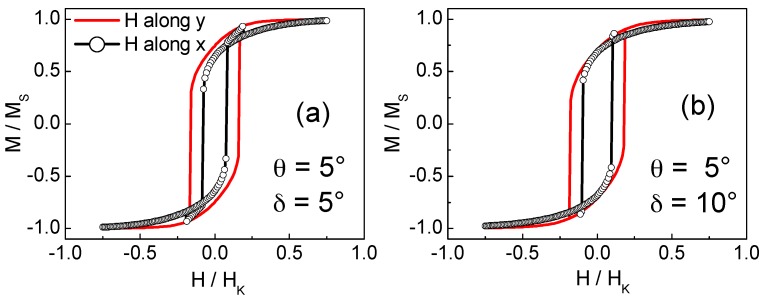
Simulated hysteresis loops obtained for θ = 5° and for the indicated values of δ, with magnetic field H applied along the y axis (line) and the x axis (line with circles).

## Appendix A

To gain a deeper insight into the hysteresis process affected by branch-crossing, let us examine in more detail the simulated loop obtained for δ = 0°, θ = 5° and the magnetic field applied along x, already shown in Figure 7b. Each point of the hysteresis loop corresponds to a specific configuration of the magnetization (magnetization map) of the system, modeled by the grid of cells in Figure 6. In Figure A1, the simulated loop is displayed (central frame) together with eight magnetization maps relative to the points indicated on the loop itself. The arrow, visible in each cell of the maps, indicates the orientation of the magnetization (as already explained above, the magnetization is supposed to be uniform in each cell). The color of each cell provides information about the projection of the magnetization along x (M_x_), namely in the direction of the applied magnetic field: blue and red designate the sign of M_x_ (positive and negative, respectively) and the color intensity is proportional to the magnitude of |M_x_|.

To mimic the experimental loop measurement procedure, our calculation of the simulated loop starts from the positive magnetic saturation state, namely from map (a) (different shades of blue characterize the matrix and the Co cluster because of the different values of M_S_alloy_ and M_S_Co_). With decreasing the applied field, i.e., moving to point (b) and then to the positive remanence state (c), the interplay between the matrix-cluster exchange coupling and the anisotropies’ strengths and directions—the misalignment θ of K_Co_ plays a particularly crucial role—favors an upward orientation of the magnetization arrows in the cells. This means that the projection of the magnetization along y (M_y_) is positive. At point (d), the negative field, applied along x, has been sufficient to reverse the largest fraction of the magnetization of the system. Hence, M_x_ < 0, whereas M_y_ is still positive, even in the Co cluster. In fact, the exchange coupling with the matrix dominates on K_Co_ that here is forcing the Co magnetization downward, because of the misalignment θ. With further decreasing the field, a magnetic configuration settles on with arrows pointing downward, which results in M_y_ < 0 (this map is not shown, but the configuration is qualitatively similar to that visible also in frame (f), along the ascending branch of the loop). A sudden jump in M/M_S_ marks this passage from M_y_ > 0 to M_y_ < 0. Then, a negative saturation is achieved at point (e), where M_y_ = 0. Point (e) marks the end of the descending branch of the loop (from positive to negative saturation).

On the ascending branch of the loop (from negative to positive saturation), point (f) is analogous to point (b). In this case, K_Co_ and the matrix-cluster exchange coupling cooperate to produce a downward orientation of the magnetization arrows (i.e., M_x_ < 0 and M_y_ < 0). Point (g) is the negative remanence state. In point (h), analogous to (d), M_x_ > 0 and M_y_ < 0. Points (d) and (f) correspond to the same negative value of the applied magnetic field. The comparison of the corresponding maps reveals that the slope of the magnetization arrows with respect to the x axis, especially those of the Co cluster, is larger in map (f) than in map (d). As a consequence, |M_x_| calculated in (f) is smaller than in (d). Similarly, points (b) and (h) correspond to the same positive field. The slope of the arrows in (b) is larger than in (h) and, hence, M_x_ in (b) is smaller than in (h).

The following general behavior emerges from the analysis of the maps: at any field within the range where the branch-crossing effect occurs in the third (first) quadrant of the loop, the slope of the Co magnetization and the slope of K_Co_ have the opposite sign along the descending (ascending) branch and have the same sign along the ascending (descending) one.

## Appendix B

In Figure A2 we show the simulations obtained for the system in Figure 6, for θ = 5° and δ = 5° and 10°. Given the results for θ = 5° and δ = 0° (Figure 7b), the branch-crossing effect is seen to reduce more and more with increasing δ (no effect is observed for δ > 10°).

If we focus the attention on the simulations presenting the branch-crossing and consider the ratio between the remanence measured along y (M_ry_) and that measured along x (M_rx_), we obtain: M_ry_/M_rx_ ~ 1.32 for δ = 0° and θ = 5° (Figure 7b); M_ry_/M_rx_ ~ 1.11 for δ = 5° and θ = 0° (Figure 7g); M_ry_/M_rx_ ~ 1.17 for δ = 5° and θ = 5° (Figure A2). Thus, the misalignment of K_alloy_ is detrimental not only for the branch-crossing effect but also for the anisotropic character of the hysteretic behavior, as it tends to smooth the difference between the easy and hard axes.
